# Dopamine D4 Receptor Activation Increases Hippocampal Gamma Oscillations by Enhancing Synchronization of Fast-Spiking Interneurons

**DOI:** 10.1371/journal.pone.0040906

**Published:** 2012-07-17

**Authors:** Richard Andersson, April Johnston, André Fisahn

**Affiliations:** Neuronal Oscillations Laboratory, KI-Alzheimer Disease Research Center, Department of Neurobiology, Care Sciences and Society, Karolinska Institute, Stockholm, Sweden; Chiba University Center for Forensic Mental Health, Japan

## Abstract

**Background:**

Gamma oscillations are electric activity patterns of the mammalian brain hypothesized to serve attention, sensory perception, working memory and memory encoding. They are disrupted or altered in schizophrenic patients with associated cognitive deficits, which persist in spite of treatment with antipsychotics. Because cognitive symptoms are a core feature of schizophrenia it is relevant to explore signaling pathways that potentially regulate gamma oscillations. Dopamine has been reported to decrease gamma oscillation power via D1-like receptors. Based on the expression pattern of D4 receptors (D4R) in hippocampus, and pharmacological effects of D4R ligands in animals, we hypothesize that they are in a position to regulate gamma oscillations as well.

**Methodology/Principal Findings:**

To address this hypothesis we use rat hippocampal slices and kainate-induced gamma oscillations. Local field potential recordings as well as intracellular recordings of pyramidal cells, fast-spiking and non-fast-spiking interneurons were carried out. We show that D4R activation with the selective ligand PD168077 increases gamma oscillation power, which can be blocked by the D4R-specific antagonist L745,870 as well as by the antipsychotic drug Clozapine. Pyramidal cells did not exhibit changes in excitatory or inhibitory synaptic current amplitudes, but inhibitory currents became more coherent with the oscillations after application of PD168077. Fast-spiking, but not non-fast spiking, interneurons, increase their action potential phase-coupling and coherence with regard to ongoing gamma oscillations in response to D4R activation. Among several possible mechanisms we found that the NMDA receptor antagonist AP5 also blocks the D4R mediated increase in gamma oscillation power.

**Conclusions/Significance:**

We conclude that D4R activation affects fast-spiking interneuron synchronization and thereby increases gamma power by an NMDA receptor-dependent mechanism. This suggests that converging deficits on fast-spiking interneurons may lead to decreased network function and thus aberrant gamma oscillations and cognitive decline in schizophrenia.

## Introduction

Gamma oscillations (rhythmic electric activity in neuronal networks at 30–80 Hz) are believed to be a mechanism for neuronal processing of information relating to memory, attention [1], working memory [2], sensory stimuli and perception [3], and are correlated with cognitive performance and memory load [4]. Moreover, gamma oscillations are also hypothesized to establish top-down control over neuronal networks [5], functionally bind them and regulate gain to improve information transfer between brain areas [6]. Gamma oscillations are typically recorded using electroencephalography (EEG) in humans and are present in the neocortex as well as the hippocampal complex, where the layered cytoarchitecture and the synchronized neuronal activity produces rhythmic local field potential (LFP) deflections [1].

This type of oscillatory activity is reliant upon fast rhythmic synaptic inhibition of the perisomatic regions of pyramidal cells. This function is served by fast-spiking parvalbumin-positive basket cells (interneurons) [7]. These neurons are highly interconnected, whereby they can self-synchronize [8], [9], [10] and, given enough excitation, can drive gamma oscillations [7]. Phasic inhibition provided by fast-spiking interneurons (FS) sets timing windows in which pyramidal cells and other neurons are able to fire action potentials. Therefore strong rhythmical inhibition also establishes rhythmical excitatory feed-back onto fast-spiking interneurons further powering the oscillatory activity [11].

Convergent evidence shows that parvalbumin-positive FS play a pivotal role in the cognitive impairments seen in schizophrenia. Several major schizophrenia risk-genes are associated with these neurons [12], [13], [14]. The number of parvalbumin-positive interneurons decreases in the hippocampi of patients with schizophrenia [15], possibly because of excitotoxic damage, which has been linked to NMDA receptor dysfunction [14], [16]. The FS exert powerful effects in the electric behavior of connected neurons [7]. It is therefore interesting that, consistent with the cognitive deficits in schizophrenia, gamma oscillations are disrupted in several areas of the brain in schizophrenic patients [17], [18], particularly the prefrontal, auditory and visual cortices as well as the hippocampus [14], [19], [20], [21]. In a recent study it was shown that the cognitive deficits arise in childhood before the onset of the full clinical symptoms of schizophrenia [22]. This may reflect a pathological process in adolescence involving the parvalbumin-positive interneurons, which contributes to the development of the disease. Thus, rather than disrupted gamma oscillations arising later in the disease as a side effect of medication, it has been hypothesized that abnormal gamma oscillations are a core pathophysiological feature of schizophrenia [12], [14], [20]. In patients, this disruption is observed as an increased “background” level of gamma oscillations but a decreased ability to dynamically respond to cognitive tasks [12].

The various aspects used to clinically define schizophrenia are typically divided into positive, negative and cognitive symptoms. Successful pharmacological treatment exists for the positive symptoms (hallucinations, delusions etc.) in the form of dopamine D2 receptor (D2R) antagonists. Still, negative and cognitive symptoms remain largely unaddressed [12], which provides an impetus to study further the pharmacology and pathophysiology of schizophrenia. The success of D2R antagonists as therapeutic agents led to the “dopamine hypothesis” of schizophrenia with the core assumption that excessive dopamine levels lead to psychosis. One of the most efficacious antipsychotic drugs is Clozapine. It is a ligand on a variety of G-protein coupled receptors and is a more potent antagonist on D4R than on D2R [23]. As D4R is predominately expressed in areas such as the prefrontal cortex, entorhinal cortex dorsal and ventral striatum and the hippocampal complex it has been suggested to have a role in regulating cognitive function [24], [25], [26]. It is mostly expressed in interneurons [27], but there may also be expression in pyramidal cells in cortical areas [28]. Studies in cultured prefrontal neurons show that D4R might be involved in regulating the cell membrane expression of GABA_A_- and NMDA receptors in pyramidal cells [29], [30] and AMPA receptors in interneurons [31]. Because of the pharmacological profile of Clozapine, D4R have been suggested to carry some of the beneficial effects that set clozapine apart from traditional high affinity D2 receptor antagonists in the treatment of schizophrenia [32]. Selective D4R antagonists did not however produce an antipsychotic effect in clinical trials [33], [34], [35], demonstrating that this receptor is functionally distinct from D2/3R.

Based on the areas where D4R is reported to be expressed we believe that it is relevant to investigate whether this receptor has a role in regulating physiological activity relevant to cognition. We aimed to shed light on the effects of D4R activation on gamma oscillations in the hippocampus. We also wanted to assess the possibility whether the ionotropic receptors involved in D4R-mediated modulation reported in the prefrontal cortex are involved in the effects observed in the hippocampus [31].

## Results

### D4 Receptor Activation Augments Hippocampal Gamma Oscillations

**Figure 1:**
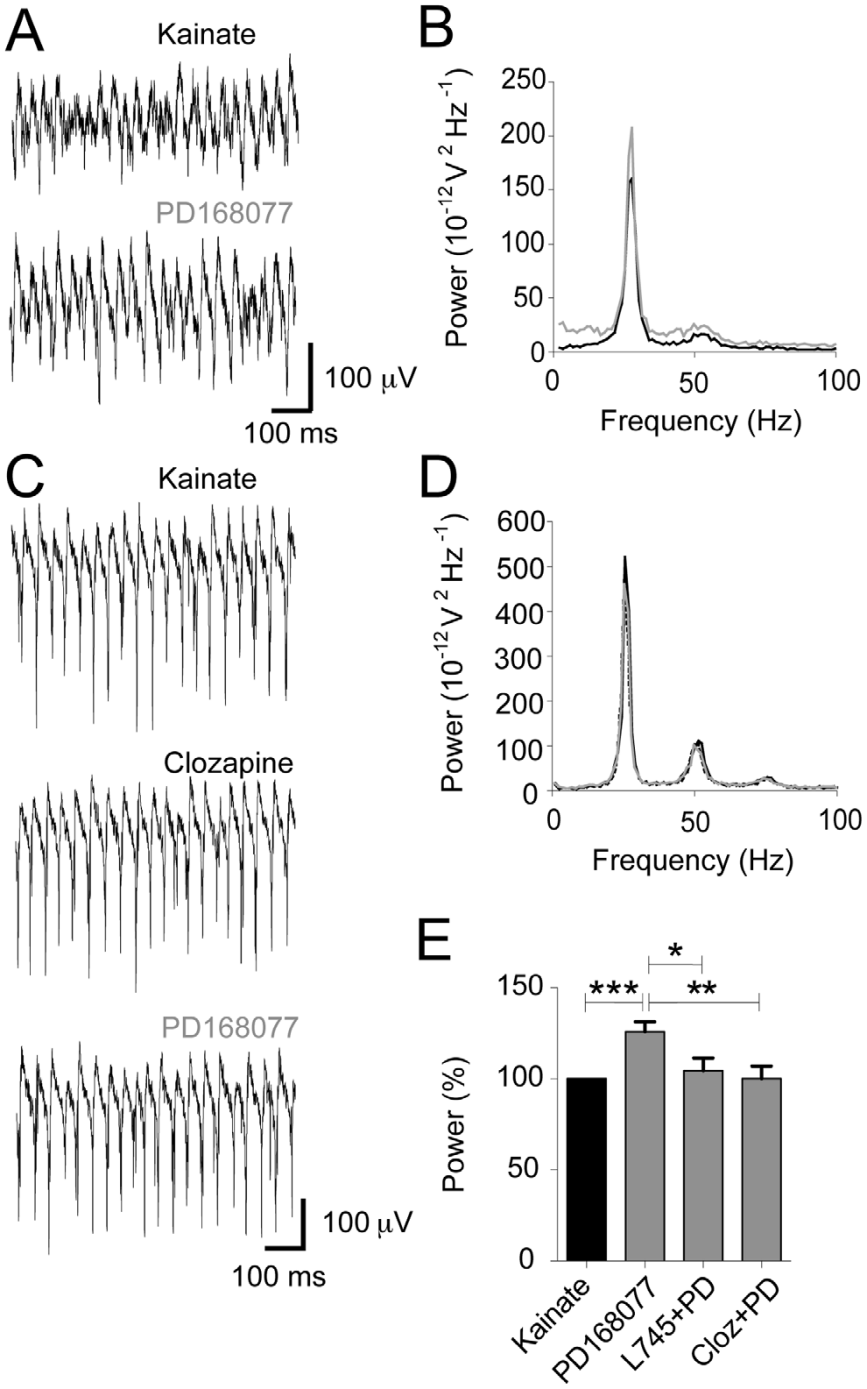
D4 receptor activation increases gamma oscillation power. LFP recordings of kainate-induced gamma oscillation and dopamine D4R agonist (PD168077, 100 nM) without and with prior application of D4R antagonist (L745,870, 500 nM or Clozapine (2 µM). **A.** Example traces of LFP recordings, showing an increase with D4R activation **B.** Power spectra of traces shown in A (kainate recording in black, subsequent recording with PD168077 in grey). **C.** Example traces of LFP recordings, showing the D4 effect is blocked with application of clozapine. **D.** Power spectra of traces shown in C. (kainate recording in black, clozapine as dotted line, and PD168077 in grey). **E.** Summary bar diagram of power (in % relative to initial kainate power, mean ± SEM). D4R activation significantly increases LFP gamma power, which is prevented by prior application of D4R antagonist or Clozapine. * P<0.05, ** P<0.01, *** P<0.001 (in unpaired t-tests).

In order to examine the effects of D4R activation on gamma oscillations in the hippocampal complex, we used the *in vitro* model of gamma oscillations [36]. Its advantages are the induction of stable oscillations and the ease of both drug application and cell visualization. Application of 100 nM kainate reliably produced gamma oscillations in hippocampal area CA3 (30.86 Hz ±0.51 Hz, n = 55) [37], [38]. Extracellular application of the D4R agonist PD168077 (100 nM) produced an increase in gamma oscillation power (Fig. 1A, B, KA: 2.90×10^−9^±6.85×10^−10^ V^2^, PD168077: 4.45×10^−9^±1.39×10^−9^ V^2^, n = 55, Wilcoxon test p<0.001). This effect could be blocked by the specific D4R antagonist L745,870 (Fig. 1E, KA: 6.48×10^−10^±2.32×10^−10^ V^2^, L745,870: 1.57×10^−9^±5.39×10^−10^ V^2^, PD168077: 1.51×10^−9^±5.48×10^−10^ V^2^, n = 17, Friedman test: F_2,38_ = 1.06, p = 0.59). The effects of PD168077 on gamma oscillations were also blocked in the presence of Clozapine (2 µM, Fig. 1C–E, KA: 2.44×10^−9^±4.87×10^−10^ V^2^, Clozapine: 2.41×10^−9^±4.84×10^−10^ V^2^, PD168077: 2.52×10^−9^±5.91×10^−10^ V^2^, n = 10, Friedman test: F_2,18_ = 0.60, p = 0.83), which is a widely used antipsychotic drug and an antagonist on D4R [23].

### EPSC Characteristics in Pyramidal Cells are Unaffected by D4 Receptor Activation

**Figure 2:**
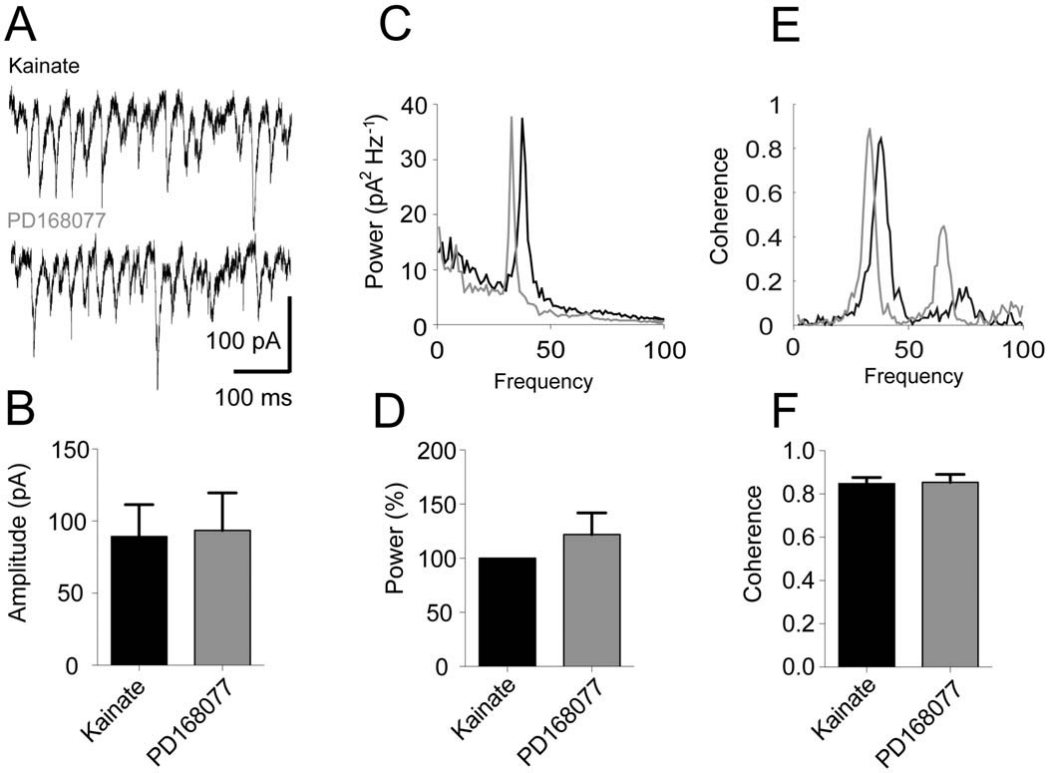
Pyramidal cell EPSCs are unaffected by D4 receptor activation. Intracellular recordings of EPSCs in pyramidal cells (clamped to −70 mV) with ongoing gamma oscillations. **A.** Example traces of EPSC recordings. **B.** Summary bar-diagram showing the amplitude of EPSCs (mean ± SEM). **C.** Power spectrum of EPSCs constructed from the traces shown in A (kainate recording in black, subsequent recording with PD168077 in grey). **D.** Summary bar-diagram of power normalized to kainate. **E.** Coherence spectrum of EPSCs versus LFP recordings from the traces shown in A (kainate recording in black, subsequent recording with PD168077 in grey). **F.** Summary bar-diagram of peak coherence values across experiments (mean ± SEM).

As synaptic currents in pyramidal cells have a substantial impact on the power of gamma oscillations, we proceeded to examine the potential effects of D4R activation on the excitatory and inhibitory synaptic currents in pyramidal cells. Concomitant recordings of both Local Field Potential (LFP) oscillations and excitatory post synaptic currents (EPSCs, recorded at −70 mV) revealed no differences in amplitude of the synaptic currents nor the number of detected events (Fig. 2A, B, KA: 89.3±22.1 pA, PD168077: 93.6±26.0 pA, n = 11, Wilcoxon test: p = 0.52, KA: 29.1±1.6 s^−1^ events, PD168077 29.3±1.4 s^−1^, n = 11, paired t-test: p = 0.89). The rhythmical pattern of EPSCs in pyramidal cells during ongoing gamma oscillations is clear from the example traces in Fig. 2A. Indeed, Fourier analysis of these recordings reveals clear and narrow gamma-frequency peaks in power spectra (Fig. 2C). However, EPSC power remained unchanged in response to D4R activation (KA: 1098±651 pA^2^. PD168077: 1499±1090 pA^2^, n = 11, Wilcoxon test: p = 0.64), and when each recording was normalized to the initial power after kainate application, average EPSC power after PD168077 addition was non-significant (122% ±20% Fig. 2D).

Changes in the timing of EPSCs with regard to the LFP oscillation are another possible mechanism that could explain the increase of LFP power in response to D4R activation. However, no significant differences were found in the coherence between EPSCs and LFP oscillations before and after D4R activation, suggesting no change in excitatory input onto pyramidal cells (Fig. 2E example coherence spectrum with a peak in the gamma-frequency range, Fig. 2F peak coherence across experiments KA: 0.85±0.03, PD168077: 0.85±0.04, n = 11, paired t-test: p = 0.88).

### D4 Receptor Activation Increases Coherence of IPSCs in Pyramidal Cells

**Figure 3:**
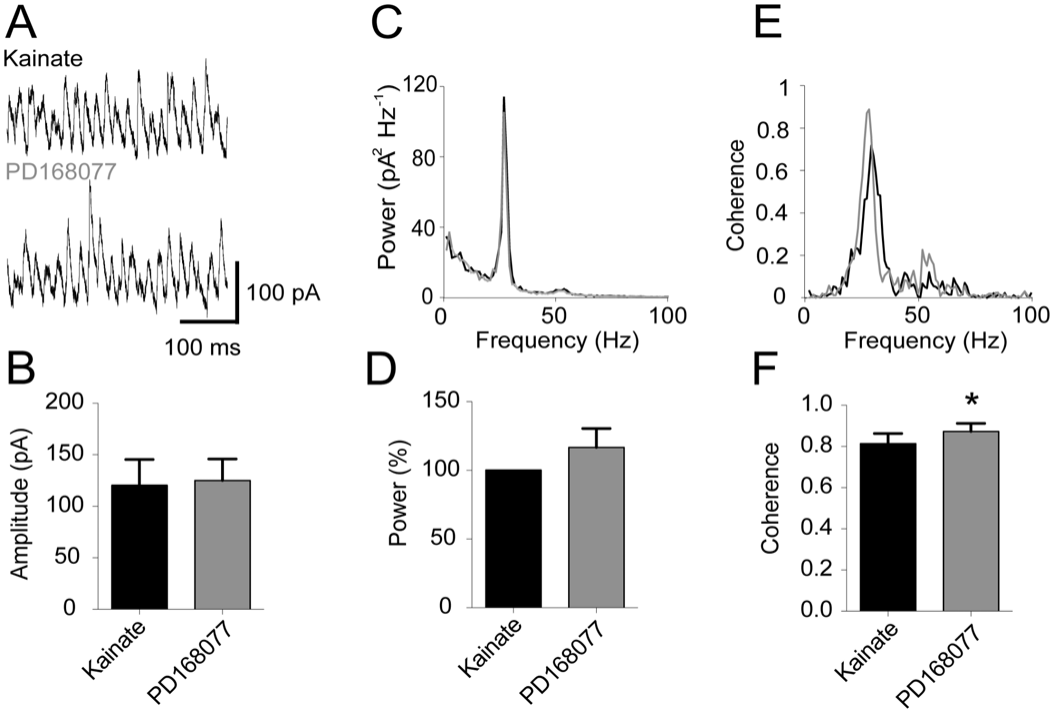
D4 receptor activation increases coherence of pyramidal cell IPSCs. Intracellular recordings of IPSCs in pyramidal cells (clamped to 0 mV) with ongoing gamma oscillations. **A.** Example traces of IPSC recordings. **B.** Summary bar-diagram showing the amplitude of IPSCs (mean ± SEM). **C.** Power spectrum of IPSCs constructed from the traces shown in A. (kainate recording in black, subsequent recording with PD168077 in grey). **D.** Summary bar-diagram of LFP gamma power normalized to kainate. **E.** Coherence spectrum of IPSCs versus LFP recordings from recordings shown in A (kainate recording in black, subsequent recording with PD168077 in grey). **F.** Summary bar-diagram of peak coherence values across experiments (mean ± SEM).

As we did not observe any systematic change in the EPSCs we next turned to consider the inhibitory postsynaptic currents in pyramidal cells. Inhibitory synaptic activity contributes substantially to shaping the voltage deflections in the LFP oscillations [39]. Moreover, D4R activation in cultured prefrontal cortex pyramidal cells was shown to reduce GABA_A_ receptor-mediated currents [29]. We therefore wanted to test the hypothesis that alterations in IPSC amplitude, their power or coherence, could underlie or contribute to the increase in gamma oscillation power produced by PD168077. To this end, concomitant voltage clamp and LFP recordings were carried out. Pyramidal cells were held at 0 mV for the duration of the recording (Fig. 3A). As was the case for EPSCs, the IPSC amplitude remained unaffected by D4R activation (Fig. 3B, KA: 120.4±25.0 pA, PD168077: 125.1±20.8 pA, n = 9, Wilcoxon test: p = 0.57). This was also the case for the rate of IPSCs (KA: 25.3±1.7 s^−1^, PD168077: 25.2±1.5 s^−1^, n = 9, paired t-test: p = 0.86). IPSCs were also rhythmic with clear power spectral peaks in the gamma-frequency range (Fig. 3C) but the power of IPSCs likewise remained unchanged in response to D4R activation (KA: 1350±590 pA^2^, PD168077: 1310±490 pA^2^, n = 9, Wilcoxon test: p = 0.99). When each recording was normalized to the initial power after kainate application, average IPSC power after PD168077 application was non-significant (117% ±13.8% Fig. 3D). In contrast to what we found for EPSCs, a significant increase in coherence was found for IPSCs (Fig. 3E, F, KA: 0.81±0.05, PD168077: 0.87±0.04, n = 9, paired t-test, p<0.05) indicating that the level of interneuron synchronization may play a role in the D4R-mediated increase of hippocampal gamma oscillations.

### Action Potential Discharge in Pyramidal Cells is Unaffected by D4 Receptor Activation

**Figure 4:**
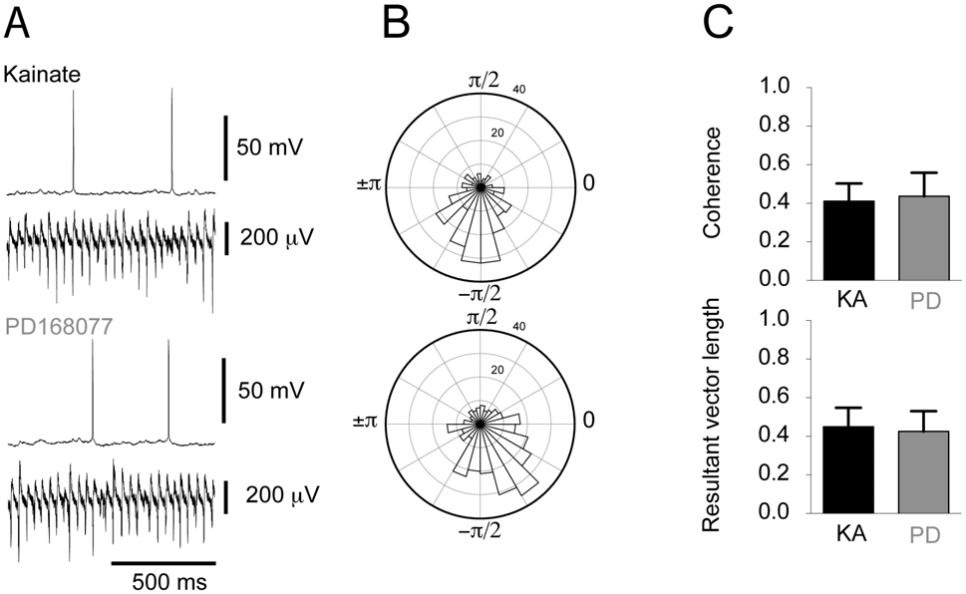
Spike-phase coupling in pyramidal cells is unaffected by D4 receptor activation. **A**. Example traces of concomitant recordings of a pyramidal cell and LFP oscillations before and after the addition of PD168077. **B**. Circular histograms based on recordings shown in A, indicating the number of action potentials discharged in each phase from −π to π. The radial axis indicates the number of action potentials. The higher and lower histograms represent recordings before and after PD168077 application, respectively. **C**. Summary bar-diagrams representing mean peak coherence and resultant vector length, respectively (mean ± SEM; kainate recording in black, subsequent recording with PD168077 in grey).

As the EPSC and IPSC amplitudes did not change, it appeared as if direct modulation of neither of the post synaptic currents in pyramidal cells was the mechanism driving D4R-mediated increase in LFP gamma power. In an earlier study we found that spike-phase coupling and/or phase synchrony of pyramidal cells were crucial factors in the histaminergic modulation of *in vitro* gamma oscillations [38]. We therefore turned our attention to the spiking patterns of pyramidal cells during ongoing LFP oscillations before and after D4R activation. Concomitant current clamp recordings of pyramidal cells and LFP gamma oscillations were carried out (Fig. 4A). The mean resting membrane potential of pyramidal cells was not altered by PD168077 application (KA: −48.56±1.03 mV, PD168077: −48.74±0.84 mV, n = 7; paired t-test: p = 0.79). The instantaneous phase of the action potentials *versus* the LFP recording was calculated using the Hilbert transform and used to determine in which phase the majority of the pyramidal cell discharge occurs (Fig. 4B). No change in the overall number of action potentials was observed after D4R activation (KA: 3.12±0.56 s^−1^, PD168077: 2.83±0.77 s^−1^, n = 7, Wilcoxon, p = 0.53). Next we tested whether D4R activation affected the phase coupling of action potentials to the LFP oscillation. Measurements of phase angle, resultant vector (Fig. 4C, KA: 0.45±0.10, PD168077: 0.43±0.11, n = 7, paired t-test: p = 0.55) and preferred angle (Table S1), as well as coherence (Fig. 4C, KA: 0.41±0.09, PD168077: 0.44±0.12, paired t-test: p = 0.64) were used to establish that pyramidal cell firing behaviour remains unaltered in response to D4R activation.

### D4 Receptor Activation Increases Coherence of Fast-spiking Interneuron Activity

As pyramidal cell firing did not change with D4R activation, but inhibitory postsynaptic currents became more coherent, we hypothesized that inhibitory interneuron action potential discharge was rendered more phase-synchronized with the gamma oscillations. To investigate this notion, we proceeded to assess the firing patterns of interneurons. There are several methods to classify interneurons using anatomical, electrophysiological, biochemical/molecular and developmental profiles. For complete identification of interneuron classes it is necessary to use all of the aforementioned measures with a large number of neurons and then sort the neurons through a clustering algorithm [40]. For the purpose of this study, we chose to distinguish between fast-spiking interneurons (FS) and non-fast-spiking interneurons (nFS) by electrophysiological means (Figure S1) in order to establish whether one or the other interneuron subgroup is associated with the D4R effect.

**Figure 5:**
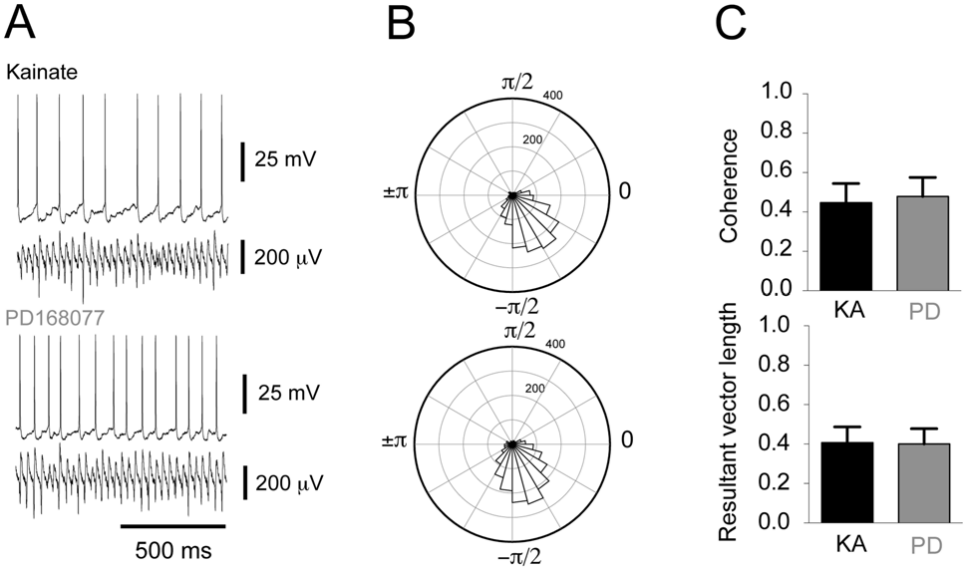
Spike-phase coupling in non-fast spiking interneurons is unaffected by D4 receptor activation. **A**. Example traces of concomitant recordings of non-fast spiking interneurons and LFP oscillations before and after the addition of PD168077. **B**. Circular histograms based on recordings similar to those in A, indicating the number of action potentials discharged in each phase from −π to π. The radial axis indicates the number of action potentials. The higher and lower histograms represent recordings before and after PD168077 application, respectively. **C**. Summary bar-diagrams representing mean peak coherence and resultant vector length, respectively (mean ± SEM; kainate recording in black, subsequent recording with PD168077 in grey).

We conducted concomitant recordings of nFS spiking and LFP gamma oscillations. The nFS were less depolarized at rest than pyramidal cells and were not depolarized by D4R activation (Fig. 5A, KA: −51.08±1.21 mV, PD168077: −49.95±1.36 mV, n = 13, paired t-test p = 0.19). As described for pyramidal cells, we constructed circular histograms for nFS action potential discharge patterns during ongoing gamma oscillations (Fig. 5B). No change was observed in the firing rate of nFS after D4R activation (KA: 16.82±2.42 s^−1^, PD168077: 18.30±2.46 s^−1^, n = 13 paired t-test: p = 0.25), or in spike-phase coupling (Fig. 5C; KA: 0.41±0.08, PD168077: 0.40±0.08, n = 13, paired t-test: p = 0.82), coherence (Fig. 5C; KA: 0.45±0.10, PD168077: 0.48±0.10, n = 13, paired t-test: p = 0.17) and preferred angle (Table S1).

**Figure 6:**
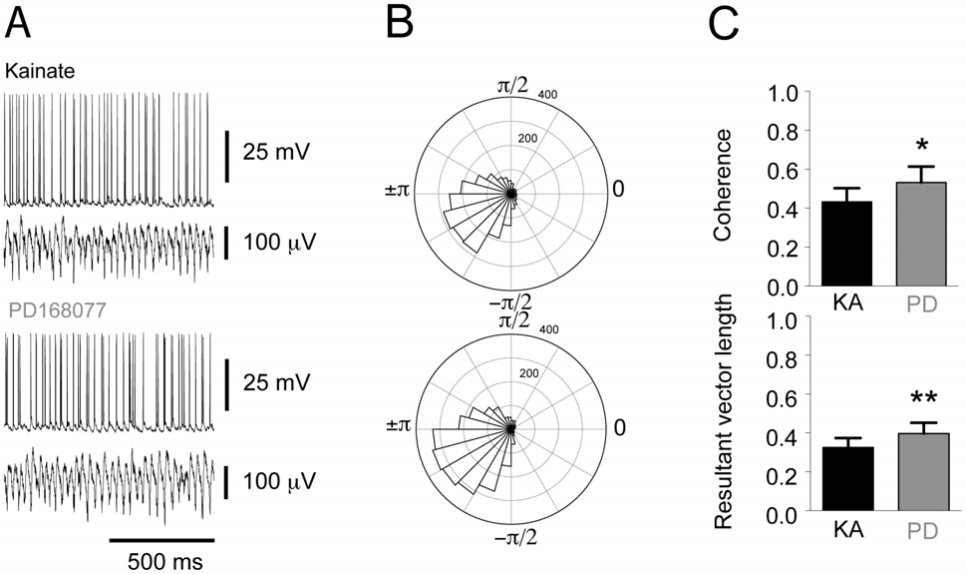
D4 receptor activation increases spike-phase coupling in fast-spiking interneurons. **A**. Example traces of concomitant recordings of fast spiking interneurons and LFP oscillations before and after the addition of PD168077. **B**. Circular histograms based on recordings shown in A, indicating the number of action potentials discharged in each phase from −π to π. The radial axis indicates the number of action potentials. The higher and lower histograms represent recordings before and after PD168077 application, respectively. **C**. Summary bar-diagrams representing mean peak coherence and resultant vector length, respectively (mean ± SEM, * indicates p<0.05, ** p<0.01; kainate recording in black, subsequent recording with PD168077 in grey).

Next we examined the spiking patterns of FS interneurons. Concomitant intracellular and LFP recordings revealed an unchanged resting membrane potential (Fig. 6A, KA: −52.91±1.11 mV PD168077: −52.48±1.20 mV, n = 14, paired t-test: p = 0.30) and average spike rate in response to D4R activation (Fig. 6B, KA: 18.73±3.20 s^−1^, PD168077: 17.33±3.20 s^−1^, n = 14, paired t-test: p = 0.42). The circular statistical analysis indicates that the preferred phase-angle of action potential firing remained unaffected by D4R activation (Table S1). In contrast to nFS interneurons, action potential discharge in FS interneurons is more concentrated around the preferred phase-angle after D4R activation, and the coherence between membrane potential fluctuations and LFP is increased (Fig. 6C; Resultant vector length: KA: 0.33±0.05, PD168077: 0.40±0.05, n = 14, paired t-test: p<0.01; Coherence: KA: 0.43±0.07, PD168077: 0.53±0.08, n = 14, paired t-test: p = 0.02).

### AMPA Receptor-mediated Currents in Fast-spiking Interneurons are Unaffected by D4 Receptor Activation

**Figure 7:**
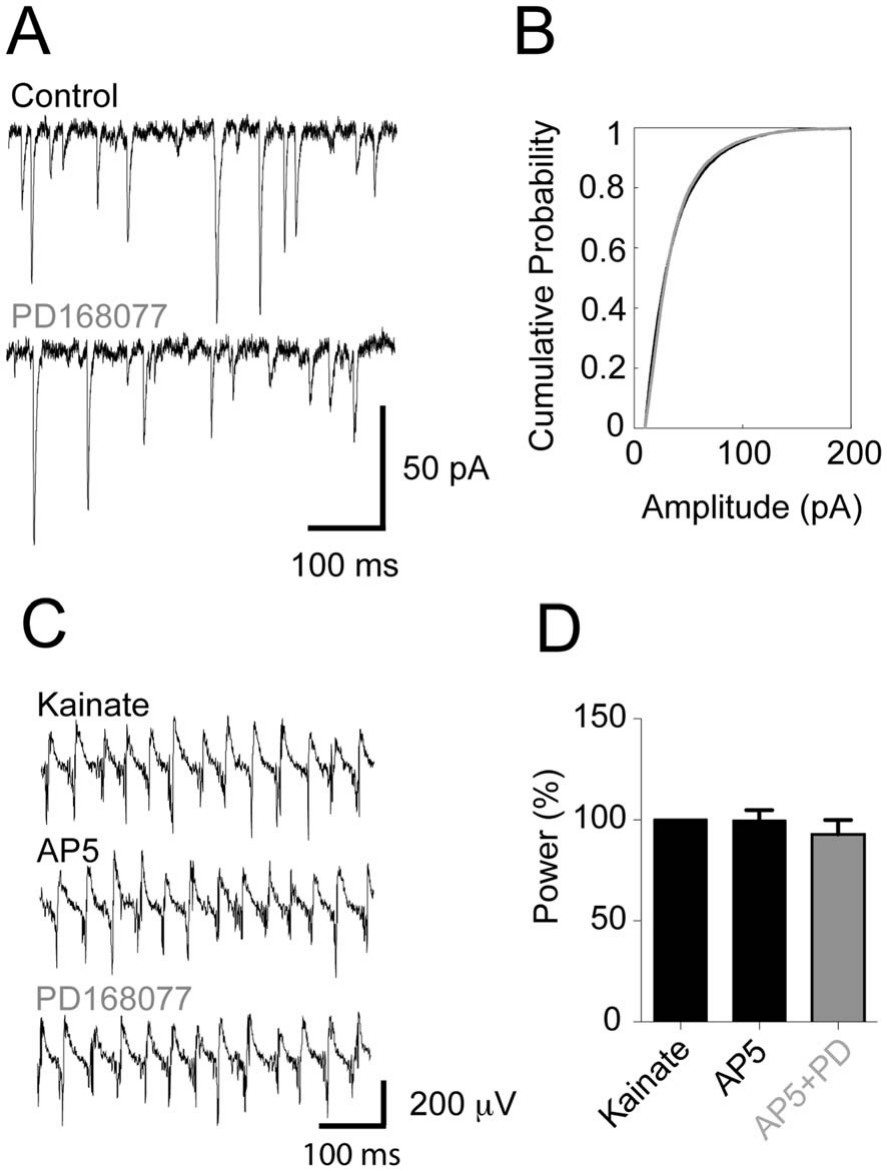
D4 receptor modulation of gamma oscillations is NMDAR-dependent. EPSC recordings recorded from fast-spiking interneurons and the effect of NMDA receptor antagonists on LFP gamma power modulation by PD168077 **A**. Example traces of EPSCs (clamped to −70 mV, 50 µM picrotoxin, 50 µM AP5). **B**. Aggregated amplitudes across experiments plotted in an empirical cumulative distribution function (kainate recording in black, subsequent recording with PD168077 in grey). Note that the 2 graphs overlap to a large extent. **C.** Example traces of LFP recordings showing oscillations from (top to bottom) KA, the addition of AP5, the addition of PD 168077. **D.** Summary bar-diagram of LFP gamma power (in % relative to initial kainate power, means ± SEM). Addition of NMDA receptor antagonist AP5 does not have an effect on the gamma power but completely blocks the increase produced by D4R activation.

With FS interneurons as the only cell class directly sensitive to D4R activation we wanted to test the hypothesis that D4R might regulate AMPA receptor currents in hippocampal interneurons in a mechanism similar to what has been reported for prefrontal cortex interneurons [31]. Hence we proceeded to record spontaneous EPSCs in these neurons (in the absence of kainate-induced gamma oscillations). Intracellular recordings of FS interneurons (held at −70 mV), in the presence of 50 µM AP5 and 50 µM picrotoxin, yielded clear and numerous EPSCs (Fig. 7A). There was no significant difference in the number of events before and after D4R activation (control: 22.9±4.1 s^−1^, PD168077: 25.6±5.5 s^−1^, n = 6, paired t-test: p = 0.56). Moreover, the distribution of aggregated EPSC amplitudes from these experiments were found to be overlapping in an empirical cumulative distribution plot (Fig. 7B), indicating that there is no change in amplitude distribution in response to D4R activation (mean EPSC amplitudes: KA: 35.30±5.7 pA, PD: 36.3±4.9 pA, n = 6, paired t-test: p = 0.56 [41]). It should be noted however that while NMDA receptors were blocked with AP5 the EPSCs we recorded are likely composed of both AMPA and Kainate receptor- (KAR) mediated currents. KAR-mediated current amplitude, however, is very small in comparison to current amplitudes mediated by AMPA receptors. Additionally, the kinetics of KAR are slower than those of AMPA receptors [42]. We therefore speculated that while KAR may not contribute to the amplitude of the EPSCs, a hypothetical increase in KAR conductance or membrane expression in response to D4R activation may extend the duration of the EPSCs. However, we did not find any difference in the half-width of the EPSCs to support this theory (control: 3.82±0.72 ms, PD168077: 3.30±0.50 ms, n = 6, paired t-test p = 0.36).

### NMDA Receptor Antagonist Blocks D4 Receptor-mediated Increase of Gamma Oscillation Power

It has been suggested that reduced NMDAR currents in fast-spiking interneurons are responsible for disturbances of gamma oscillations and cognitive decline [43], [44]. Moreover, it has also been reported that D4 receptors regulate NMDA receptor currents in *stratum oriens* of CA1 [45]. We therefore examined the role of NMDAR currents in the D4R-mediated effect on gamma oscillations. To test whether NMDAR on FS interneurons may have a role in the D4R-mediated increase of gamma oscillation power we recorded LFP gamma oscillations after D4R activation in the presence of a NMDAR antagonist (Fig. 7C, 50 µM AP5). While addition of AP5 did not alter the kainate-induced gamma oscillations (KA: 1.43×10^−9^±3.17×10^−10^ V^2^, AP5: 1.33×10^−9^±2.70×10^−10^ V^2^) it prevented the D4R-mediated increase in power after subsequent addition of PD168077 (PD168077: 1.13×10^−9^±2.31×10^−10^ V^2^, n = 14 Friedman test: F_2,26_ = 1.782, p = 0.41). We also analysed the power when normalized to kainate (Fig. 7D, AP5: 99.74±5.07%, PD168077: 92.80±7.12%, n = 14 paired t-test: p = 0.47).

### D4 Receptor Activation Decreases Outward Current in Fast-spiking Interneurons

**Figure 8:**
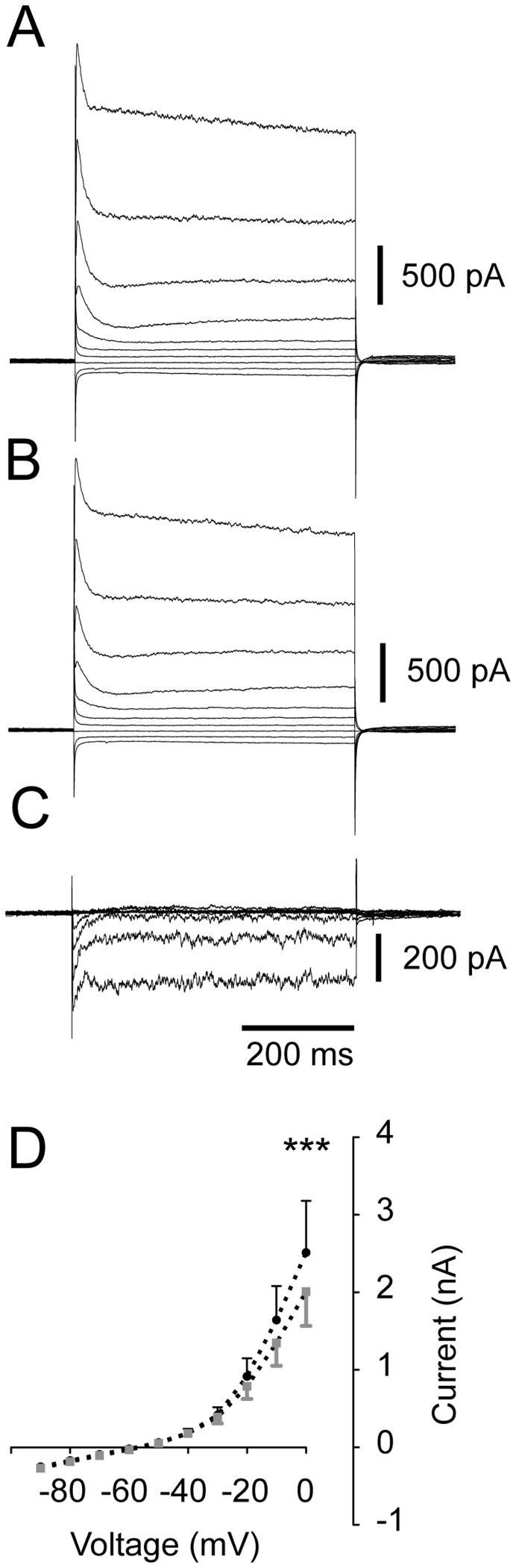
D4 receptor activation causes reduction in high-voltage outward current in fast-spiking interneurons. Voltage steps recorded in fast-spiking interneurons (with 1 µM TTX present, 10 mV steps from −90 mV to 0 mV). **A.** Traces of current responses in control conditions (no kainate). **B.** Traces of current responses in the presence of PD168077. **C**. Traces of differences (digital subtraction of trace in B-A). **D**. Summary current vs. voltage plot across experiments. Black represents control, grey PD168077. *** P<0.001 (two-way ANOVA).

Potassium currents (subject to G-protein coupled receptor regulation) have been shown to regulate pyramidal cell firing patterns that have a strong impact on gamma oscillations [46]. We therefore considered the possibility of finding a difference in the amplitude of outward voltage gated currents when PD168077 was applied. We examined FS interneuron cellular currents across a wide voltage range. With action potentials blocked (1 µM TTX), undisturbed outward currents at higher voltage steps were recorded (Fig. 8A). Their amplitude decreased in response to D4R activation (Fig. 8B–C). This effect persisted across experiments (Fig. 8D), as in a two-way ANOVA of the voltage steps where voltage and D4R activation were indicated as factors, voltage (F_9,70_ = 14.07, PD168077 (F_1,70_ = 9.72) and the interaction between voltage and D4R activation were significant (F_9,70_ = 2.58). A Bonferroni *post-hoc* test indicated a single significant point difference between control and PD168077 at 0 mV, where the mean current amplitude is reduced with PD168077. Since under physiological conditions FS neurons will not reside in the 0 mV voltage region except during an action potential discharge we proceeded to examine action potential half-widths and after-hyper-polarization amplitudes during ongoing kainate-induced gamma oscillations. Neither parameter was significantly affected by D4R activation and as such it remains uncertain whether the difference in outward current at 0 mV is functionally relevant for the regulation of FS interneuron spike-phase coupling (action potential half-width: KA: 1.04±0.09 ms, PD168077: 1.10±0.09 ms, n = 14, paired t-test, p = 0.13; after-hyper-polarization amplitude: KA: −9.54±1.25 mV, PD168077: −9.43±1.32 mV, n = 14, paired t-test, p = 0.78).

## Discussion

Dopamine has previously been reported to decrease gamma oscillation power *in vitro* [47], [48], through a D1/5R dependent mechanism. We on the other hand found a D4R-mediated increase in gamma oscillation power. An increase in power is hypothesized to lead to increased gain of neuronal signaling [1], [6], i.e. EPSCs are more likely to trigger spikes thus integrating and propagating information more efficiently across neural networks. This can lead to increased signal-to-noise ratios in case the increased gamma oscillation power is transient but can lead to the opposite in case the increased power is persistent and irrelevant information propagates with high gain. With increased gamma oscillation background power and decreased dynamic increases in response to real cognitive need, it is possible that irrelevant and inconsistent stimuli are ascribed undue cognitive salience [12]. In the light of our findings (Fig. 1E), we speculate that this may be the case with a hyper-dopaminergic *tonus* in schizophrenia activating D4 receptors, which in turn produce an increase in “background gamma”.

In this study we show that the antipsychotic drug Clozapine can block the D4R-mediated increase in gamma oscillations, analogous to the pharmacological effect of the specific D4R antagonist L745,870 (Fig. 1E). The fact that the D4R antagonists were ineffective as antipsychotic drugs in clinical trials lead to a reinforced conclusion that the main antipsychotic effect is carried by the D2R, Our results, on the other hand, show that it is possible to modulate gamma oscillations *in vitro* by regulating D4R activity, potentially restoring the physiological information processing of neural networks. Thus, it might be of interest to investigate D4R ligands as adjuvant treatment to standard D2R antagonist antipsychotics in future clinical studies.

It is also of interest to explore the potential cellular mechanisms underlying the D4R-mediated increase in gamma oscillations power. Postsynaptic potentials in pyramidal cells contribute substantially to the Local Field Potential (LFP) deflections making up the oscillation [36], [39]. For this reason we explored the possibility of D4R-mediated modulation of EPSCs or IPSCs in pyramidal cells. Consistent with the fact that there was an absence of a systematic difference in coherence in EPSCs in pyramidal cells (Fig. 2E, F) there was no change in pyramidal cell spike-phase coupling (as assessed by the resultant vector length, Fig. 4C). IPSCs and pyramidal cell action potential discharge carries more weight in influencing the LFP than the EPSCs [39]. We therefore reasoned that that this is a potential mechanism by which gamma oscillation power can be modulated. Studies carried out in basal ganglia medium spiny neurons [49] and prefrontal cortex pyramidal cells [29] showed a decrease in IPSC amplitude after D4R activation. The underlying locus of this effect seems to be postsynaptic and a decrease in membrane expression of GABA_A_ receptors has been put forth as the mechanism in pyramidal cells [29]. GABA_A_ receptor function is crucial as blocking these receptors abolishes gamma oscillations [37]. In our study the IPSCs in pyramidal cells did not show a decrease in amplitude, but rather their coherence increased (Fig. 3E), which is consistent with the increase in spike-phase coupling in FS (Fig. 6B, C).

In previous studies we and others found that phase-desynchronization of pyramidal cell discharge in the hippocampus during gamma oscillations decreases their power [38], [46], [50]. We therefore wanted to test the converse hypothesis - that increased gamma oscillation power could be driven by increased pyramidal cell firing synchrony. That mechanism did not seem to be present in this case as pyramidal cell action potential discharge phase-coupling and coherence remained unchanged (Fig. 4B, C).

The nFS comprise several different cell types and the number of recordings in this study is not sufficient to discern the interneuron subtypes within the broader category of nFS, However, this was not our aim. As a broad category however, the nFS were not subject to spike-phase coupling changes with PD168077 (Fig. 5B, C). Increases in discharge rates as well as increases in spike-phase coupling has been identified as mechanisms underlying increases in gamma oscillation power *in vivo* [51]. None of the neuron classes in this study exhibited increased discharge-rates. The FS interneurons exhibited an increase in spike-phase coupling and coherence with the LFP oscillations (Fig. 6B, C). Based on this modulation of their activity by D4R agonists we hypothesize that the FS is the subpopulation of neurons which is responsible for the increase in gamma oscillation power.

As GABA_A_ receptor modulation in pyramidal cells did not seem to be present in our study we considered what the case would be if such modulation was present in FS. These interneurons are crucial for enforcing the network synchrony needed for gamma oscillations, hence small changes in FS electrical properties may produce substantial effects in the network oscillations. This fact has been illustrated by studies showing that changes in conductance of GABA_A_ receptors in FS produce frequency shifts in the LFP oscillations [41] and shifts in the phase angle of rhythmical FS firing [9]. As we observed neither a substantial LFP oscillation frequency shift (Figure 1A,B) nor shifts in FS, nFS or pyramidal cell preferred firing angle (Table S1), we do not have any data to support a mechanism involving regulation of GABA_A_ receptor currents in FS.

We considered modulation of excitatory synaptic transmission onto FS interneurons as this is a potential mechanism subject to dysregulation in schizophrenia [16]. Reports from the prefrontal cortex have indicated that PD168077 applied to parvalbumin-positive interneurons decreases surface expression of AMPA receptors [31]. We could not detect any effects on largely AMPA receptor-mediated EPSC amplitudes nor number of events in our experiments (Fig. 7B). The EPSCs were also likely contributed to by kainate receptor- (KAR) mediated currents. Kainic Acid (KA) was used to elicit the gamma oscillations in this study and this compound has been shown to dose-dependently increase the power of gamma oscillations [37] without causing shifts in the peak frequency of the oscillations. We did not observe any systematic changes in EPSC amplitude, half-width or the number of EPSCs per second. In a rat model of schizophrenia, FS were rendered more sensitive to KA [52]. The FS exhibited increased action potential discharge rates, shortened action potential duration, depolarized resting membrane potential and increased after-hyper-polarization amplitude. We, on the other hand, did not observe these changes in FS (Fig. 6A, B).

As the AMPAR/KAR-mediated EPSCs did not change in response to D4R activation we turned to examine the role of the NMDA receptor, which is highly implicated in the cognitive deficits in schizophrenia and is central to the glutamate hypothesis of schizophrenia as certain NMDA receptor antagonists elicit psychotic symptoms [16]. While one study has suggested that NMDA receptors on prefrontal cortex pyramidal cells are regulated by D4R [30], we instead wanted to highlight the role of NMDA receptors on hippocampal FS as these neurons altered their firing pattern in response to D4R activation. Consistent with this focus, genetic ablation of NMDA receptors specifically on parvalbumin-positive interneurons in the prefrontal cortex [44] and the hippocampus [43] has been demonstrated to produce deficiencies in cognitive function and disturbances of gamma oscillations. Applying the NMDA receptor antagonist AP5 did not change gamma oscillation power on its own but it completely abolished the increase in oscillation power produced by D4R activation with PD168077 (Fig. 7C, D). In cognitively demanding tasks, brain regions such as the prefrontal cortex, amygdala and the hippocampus, increase the power of the gamma oscillations [4]. This dynamic response to neural information processing demands is impaired in mice with specific genetic NMDA receptor ablations in parvalbumin-positive interneurons [43], [44]. Impairments of dynamic regulation of gamma oscillation power has been observed in schizophrenic patients as well [2], [12], [17]. Novel object recognition has been reported to be under the influence of D4R and NMDAR regulation [53], to which our results could perhaps serve as a mechanistic underpinning. Future studies will have to be carried out in order to test the hypothesis that it is D4R activation that increases the NMDAR-mediated currents in FS. If this assumption is correct then increased NMDAR would mean increased rhythmic excitatory feed-back onto FS as pyramidal cells discharge rhythmically [36], [54]. This, then, could explain the increased spike-phase coupling and coherence observed in FS. Blocking a substantial amount of these receptors by adding AP5 would, in line with what we observed, abolish the PD168077 effect.

D4R activation decreased an outward current (or increase an inward current), but only at very depolarized voltages (Fig. 8A–C). The difference at these voltages, which under physiological settings would only be reached during action potential discharge, did not carry over in to producing any differences in action potential half width or after-hyper-polarization amplitude. We therefore conclude that this decrease in outward current at very depolarized potentials is not a mechanism likely to account for increasing FS interneuron spike-phase coupling.

In summary, NMDA receptor dysfunction in FS has been implicated in schizophrenia [16] and disrupted gamma oscillations [43], [44]. In addition, D4R have been demonstrated to regulate long-term potentiation (LTP) through modulation of NMDA receptor currents [45]. We show that activating D4R produces an increase in gamma oscillation power that is driven by an increase in the phase-synchrony of fast-spiking interneurons while pyramidal cells and non fast-spiking interneurons remain unchanged in their spiking behavior. We show that the increase in power is NMDA receptor-dependent. Future studies should be carried out to investigate the mechanism of how NMDA receptor-mediated currents affect gamma oscillations. Rather than taking the view that schizophrenia is a consequence of a single dysregulated transmitter system such as glutamate, dopamine or GABA one should perhaps consider the proper functioning of large neuronal networks where several different mutations and transmitter imbalances produce similar results if they converge on certain vulnerable loci [13].

## Materials and Methods

### Animals

Experiments were carried out in accordance with ethical permit granted by Norra Stockholms Djurförsöksetiska Nämnd to AF (N350/10). Male Sprague-Dawley rats (postnatal days 14–23, supplied from Charles River, Germany) were used in all experiments. In brief, the animals were deeply anaesthetized using isofluorane before being sacrificed by decapitation.

### Tissue Preparation

The brain was dissected out and placed in ice-cold ACSF (artificial cerebrospinal fluid) modified for dissection. This solution contained (in mM); 80 NaCl, 24 NaHCO_3_, 25 Glucose, 1.25 NaH_2_PO_4_, 1 Ascorbic acid, 3 NaPyruvate, 2.5 KCl, 4 MgCl_2_, 0.5 CaCl_2_, 75 Sucrose. Horizontal sections (300 µm thick) of the ventral hippocampi of both hemispheres were prepared with a Leica VT1000S vibratome (Microsystems, Stockholm, Sweden). Immediately after slicing sections were transferred to a humidified holding chamber (interface-type) containing the standard ACSF. This solution contained (in mM); 124 NaCl, 30 NaHCO_3_, 10 Glucose, 1.25 NaH_2_PO_4_, 3.5 KCl, 1.5 MgCl_2_, 1.5 CaCl_2_. The chamber was held at 32°C for at least 20 minutes after dissection. It was subsequently allowed to cool to ambient room temperature (19–22°C) for a minimum of 40 minutes before transferring slices to the submerged recording chamber. The slices were continuously supplied with humidified carbogen gas (5% CO_2_, 95% O_2_).

### Drugs and Chemicals

Chemical compounds used in intracellular and extracellular solutions were obtained from Sigma-Aldrich Sweden AB (Stockholm, Sweden). Receptor ligands and other pharmacological substances were obtained from Tocris Bioscience (Bristol, UK) and Bionuclear Scandinavia (Bromma, Sweden). The following drugs were used; Kainic acid, (2*S*,3*S*,4*S*)-Carboxy-4-(1-methylethenyl)-3-pyrrolidineacetic acid, PD 168077 maleate, *N*-Methyl-4-(2-cyanophenyl)piperazinyl-3-methylbenzamide maleate, L745,870 trihydrochloride, 3-(4-[4-Chlorophenyl]piperazin-1-yl)-methyl-1*H*-pyrrolo[2,3-*b*]pyridine trihydrochloride, Picrotoxin, D-AP5, D-(-)-2-Amino-5-phosphonopentanoic acid, Tetrodotoxin (TTX).

### Electrophysiology

Recordings were carried out in hippocampal area CA3 with borosilicate glass microelectrodes, pulled to a resistance of 3–7 MΩ. Local field potentials (LFP) were recorded using microelectrodes filled with ACSF placed in Str. Pyr [36]. LFP oscillations were elicited by applying kainic acid (100 nM) to the extracellular bath [37]. In order to maintain stable LFP oscillations all recordings were performed at 32°C in a submerged chamber with a perfusion rate of 3–5 ml per minute of aerated ACSF containing 100 nM kainate. The oscillations were allowed to stabilize for 20 minutes before any recordings were carried out. Once started, the oscillations were stable for at least 1 h.

For intracellular recordings, the microelectrodes were filled with either a caesium-based intracellular solution (for IPSC recordings) containing (in mM); CsMetSO4 140, Hepes 10, MgCl2 2, EGTA 0.6, ATPNa 2, GTPNa 0.3, set to pH 7.2–7.3 with CsOH, osmolarity 270–280 mOsm or a potassium-gluconate based solution containing in mM; K-gluconate 122.5, KCl 17.5, Na_2_ATP 4, Na_2_Phosphocreatine, NaGTP, HEPES 10, EGTA 0.2, MgCl 4, set to pH 7.2–7.3 with KOH, osmolarity set to 270–280 mOsm.

Patch clamp recordings were carried out with infrared Differential Interference Contrast microscopy (ir-DIC), (Zeiss Axioskop, Germany and BX50WI Olympus, Tokyo, Japan). The physiological signals were amplified (0.1 Hz alternating current) using Axopatch 200B and Multiclamp 700B amplifiers (Molecular devices, CA, USA). LFP recordings were performed with a 4 channel amplifier/signal conditioner M102- amplifier (Electronics lab, Faculty of Mathematics and Natural Sciences, University of Cologne, Cologne, Germany) and Multiclamp 700B. The signals were sampled at 10 kHz, conditioned using a Hum Bug 50 Hz noise eliminator (Quest Scientific, North Vancouver, BC, Canada), software low-pass filtered at 1 kHz, digitized and stored using a Digidata 1322A and Clampex 9.6 software (Molecular devices, CA, USA).

During voltage clamp recordings, series-resistance was compensated 60–80% and only experiments where this resistance remained stable (change of less than 30%) throughout were used in analysis.

### Analysis

In order to minimize the impact of “between-slice” variation we used a paired statistical design throughout the study thereby focusing on the effect produced by application of the various drugs. We only used parametric tests when the data sets could be approximated to be normally distributed. The nature of statistical tests used is indicated in the relevant segments of the results section. Data in the text is reported as means ± standard errors of the means. Power spectral density plots (from 60 s long LFP recordings) were calculated in averaged Fourier-segments of 8192 points using Axograph X (Kagi, Berkeley, CA, USA). Oscillation power was calculated by integrating the power spectral density between 20 and 50 Hz. As the frequency at the peak of the power spectra of *in vitro* gamma oscillations are highly temperature dependent [38], [46], we analyzed this frequency band because the bulk gamma oscillation power lies in this range when recording at 32**°**C.

The neurons recorded from in this study were divided into one of three categories (Pyramidal cell, Fast spiking interneurons, Non-fast spiking interneurons) based on criteria encompassing morphology, and firing patterns and cellular currents. Pyramidal cells were selected based on location in Stratum Pyramidale (Str. Pyr.) and a cigar-shaped morphology. Interneurons were located in *Strata Oriens* and *Radiatum* (close to *Stratum Pyramidale*) and were separated based on electrophysiological profiles (see Methods S1 and Figure S1).

A template-based algorithm [55] incorporated in Axograph X was used to detect synaptic events (with templates chosen from the trace of interest). The coherence between synaptic events in a single neuron and the simultaneous LFP under KA induced oscillations was assessed using a custom-written MATLAB routine (Mathworks, Natick, MA, USA).

Action potentials were detected in a custom-written MATLAB routine using an amplitude threshold. For spike-phase analysis, custom-written routines in MATLAB as well as the circular statistics toolbox [56] were used. Concomitant recordings of local field potentials (LFP) and single neurons we carried out in order to relate their spiking activity to ongoing gamma band LFP oscillations.

In order to extract the instantaneous phase the LFP recording was pre-processed in MATLAB using a bidirectional phase preserving least-squares band pass filter. The pass band was set to 20–40 Hz as the power spectra for all LFP recordings with ongoing oscillations indicated that the bulk of the signal lies within this range. The instantaneous phase of the LFP oscillation was then calculated using a Hilbert transform (implemented in MATLAB) [57]. The phase in which each action potential occurred during ongoing gamma oscillations could then be defined. For mathematical expediency, and to avoid confusion with others using degrees we chose to calculate the phases in radians rather than degrees, where the trough of the oscillation cycle corresponds to −π/2 and the peak corresponds to π/2. The phase-angles and the angular “phase-coupling” of the action potentials were calculated by vector averaging. Each action potential has a phase angle and can therefore be described as a vector with length 1. We then took as a measure of phase coupling (also known as “depth of modulation” [51]) by vector averaging these phase angles. The resultant vector length extends between 0 and 1, where 0 is perfect uniform firing throughout the oscillation cycle (or possibly perfectly counter-balanced bi- or polymodal distributions) and 1 is perfect concentration of the action potentials in one and the same phase angle [51], [58]. To test whether neurons fired in a phase-related manner, all spike-phase recordings were tested for circular uniformity using Rayleigh's test. Two non-fast spiking interneurons were not phase-coupled and were excluded from further analysis, while all others had a significant phase-preference.

Apart from the resultant vector length, mean-squared coherence was used as an additional measure of the link between cellular activity and the oscillations. While the resultant vector length was used for action potentials (as they are brief and can be regarded as discrete all-or-nothing events for our purposes), mean squared coherence (referred to simple as ‘coherence’ in the text) was used for synaptic currents. The synaptic currents are entrained by the network oscillation and are therefore highly rhythmic. For this reason it was relevant to take in to account entire traces rather than discrete events. Likewise as a complement to the supra-threshold spike-phase coupling calculated as the resultant vector length, coherence takes the subthreshold intracellular membrane potential fluctuations into account.

## Supporting Information

Figure S1 **Electrophysiological characterization of non-fast spiking versus fast-spiking interneurons.** Example traces of intracellular recordings contrasting non-fast-spiking and fast-spiking interneuron spiking properties. nFS are shown to the left FS are shown to the right. **A.** Current step waveform is shown on top left. The nFS exhibits regularly spaced action potentials with slow U-shaped after-hyper-polarizations (AHPs) in response to the positive step and a characteristic “I_h_-sag” response to the negative step. The FS exhibits irregularly spaced action potentials with V-shaped AHPs. The negative step did not elicit an “I_h_-sag” but instead revealed large and frequent excitatory post synaptic potentials, which were characteristic for this interneuron-class. **B.** Current ramp waveform is shown on top left. The nFS failed to fire throughout the current ramp and the structure of the action potentials deteriorated in to small amplitude-, wide spikelets. The FS fired throughout the ramp in most cases, maintaining spike integrity in terms of width and amplitude, while retaining irregularity and high frequency firing. **C.** Current “Step-in-step” waveform is shown on top left. The initial step induces the nFS to fire at a higher frequency than baseline, the second step only causes a mild if any increase in firing frequency, with a decrease in firing frequency compared to the first step. The FS exhibits a small increases in firing frequency in response to the first step but a marked increase in firing frequency in response to the second. The FS then reverts to firing frequencies similar to that of the first step. **D.** Close-up of action potential shapes. The nFS has a slow and smooth AHP compared to the FS V-shaped AHP, which quickly recovers the membrane potential to resting levels.
(TIF)

Table S1 Action potential phase-angle is unaffected by D4 receptor activation in all neuronal classes. Using circular statistics the table shows 95% confidence intervals for mean phase angles of action potential discharge (in radians) for pyramidal cells, non-fast spiking interneurons and fast-spiking interneurons respectively. Furthermore the number of experiments per neuron class as well as corresponding p-values for Watson-Williams test for angular changes are shown.
(PDF)

Methods S1 Differentiation of Interneuron classes.
(DOCX)
