# Supplementary figures and images for: Dopamine D4 Receptor Activation Increases Hippocampal Gamma Oscillations by Enhancing Synchronization of Fast-Spiking Interneurons
Source: PLoS One. 2012 Jul 17;7(7):e40906. doi: 10.1371/journal.pone.0040906 (PMC3398948; doi:10.1371/journal.pone.0040906)

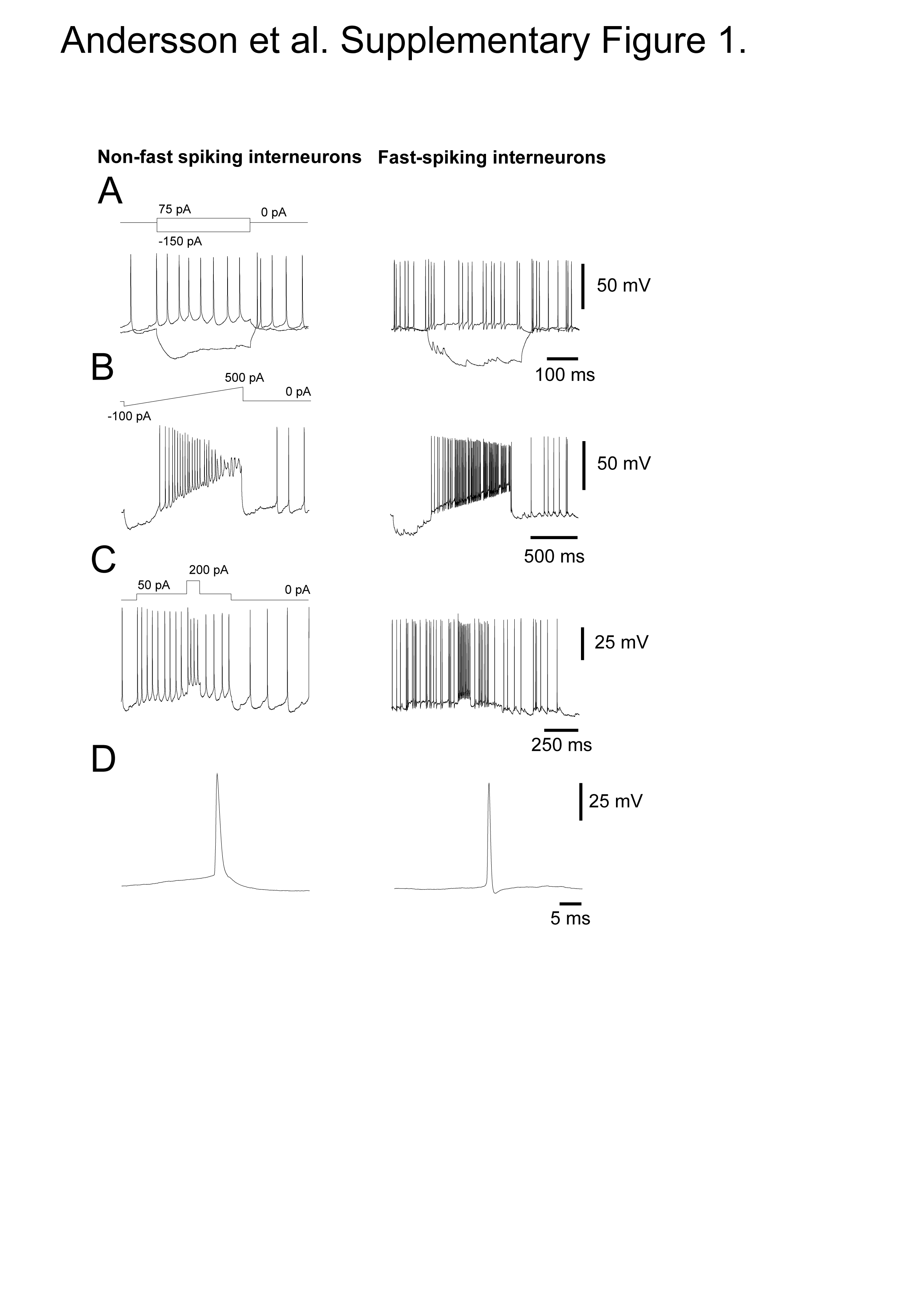

Supplement: Figure S1 — Electrophysiological characterization of non-fast spiking versus fast-spiking interneurons. Example traces of intracellular recordings contrasting non-fast-spiking and fast-spiking interneuron spiking properties. nFS are shown to the left FS are shown to the right. A. Current step waveform is shown on top left. The nFS exhibits regularly spaced action potentials with slow U-shaped after-hyper-polarizations (AHPs) in response to the positive step and a characteristic “Ih-sag” response to the negative step. The FS exhibits irregularly spaced action potentials with V-shaped AHPs. The negative step did not elicit an “Ih-sag” but instead revealed large and frequent excitatory post synaptic potentials, which were characteristic for this interneuron-class. B. Current ramp waveform is shown on top left. The nFS failed to fire throughout the current ramp and the structure of the action potentials deteriorated in to small amplitude-, wide spikelets. The FS fired throughout the ramp in most cases, maintaining spike integrity in terms of width and amplitude, while retaining irregularity and high frequency firing. C. Current “Step-in-step” waveform is shown on top left. The initial step induces the nFS to fire at a higher frequency than baseline, the second step only causes a mild if any increase in firing frequency, with a decrease in firing frequency compared to the first step. The FS exhibits a small increases in firing frequency in response to the first step but a marked increase in firing frequency in response to the second. The FS then reverts to firing frequencies similar to that of the first step. D. Close-up of action potential shapes. The nFS has a slow and smooth AHP compared to the FS V-shaped AHP, which quickly recovers the membrane potential to resting levels. (TIF) [file pone.0040906.s001.tif]
