# Supplementary material for: Dopamine D4 Receptor Activation Increases Hippocampal Gamma Oscillations by Enhancing Synchronization of Fast-Spiking Interneurons
Source: PLoS One. 2012 Jul 17;7(7):e40906. doi: 10.1371/journal.pone.0040906 (PMC3398948; doi:10.1371/journal.pone.0040906)
Supplement: Table S1 — Action potential phase-angle is unaffected by D4 receptor activation in all neuronal classes. Using circular statistics the table shows 95% confidence intervals for mean phase angles of action potential discharge (in radians) for pyramidal cells, non-fast spiking interneurons and fast-spiking interneurons respectively. Furthermore the number of experiments per neuron class as well as corresponding p-values for Watson-Williams test for angular changes are shown. (PDF) [file pone.0040906.s002.pdf]

Andersson et al.
Supplementary Table 1.

Neuron class	Treatment	lower limit	mean	upper limit	n	p
Pyramidal cell	Kainate	-2.1236	-1.2755	-0.4274	7	
	PD168077	-2.0573	-0.978	0.1013	7	0.6133
nFS	Kainate	-1.9971	-1.7301	-1.4632	13	
	PD168077	-2.2655	-1.901	-1.5365	13	0.3794
FS	Kainate	-2.16	-1.78	-1.4	14	
	PD168077	-2.27	-1.89	-1.5	14	0.67
