# Supplementary material for: Dopamine D4 Receptor Activation Increases Hippocampal Gamma Oscillations by Enhancing Synchronization of Fast-Spiking Interneurons
Source: PLoS One. 2012 Jul 17;7(7):e40906. doi: 10.1371/journal.pone.0040906 (PMC3398948; doi:10.1371/journal.pone.0040906)
Supplement: Methods S1 — Differentiation of Interneuron classes. (DOCX) [file pone.0040906.s003.docx]

**Supplementary Methods**

***Differentiation of Interneuron classes***

In order to distinguish between FS and nFS interneurons we used a series of current steps and ramp protocols. In supplementary Fig. 1A we show the responses of two representative neurons of the two classes of interneurons to positive and negative current steps. The negative step (Supplementary Fig. 1A, *left*) produced a slow hyperpolarization-activated inward current resulting in a characteristic “I_h_-sag”. This behaviour was rare in the FS neurons (supplementary Fig. 1A, *right*). In the depolarizing step the FS cells exhibited a more “stuttering” firing pattern (i.e. action potential discharge takes place in small groups followed by intermittent pauses). The nFS neurons however fired in regular intervals. We also used current ramps where the neurons also exhibited differences in their spiking patterns. The nFS cells failed to fire throughout the full extent of ramp (Supplementary Fig. 1B, *left*). The FS neurons however could in most cases maintain firing throughout the ramp (Supplementary Fig. 1B, *right*). The clearest differences however became apparent when we used a step within a step protocol. The FS can dynamically shift their firing frequencies to reflect the amount of depolarization. They therefore tended to fire at high frequencies during the “step-in-step” protocol (Supplementary Fig. 1C, *right*). The nFS neuron in contrast is less dynamic in its response and on many occasions exhibits accommodation (Supplementary Fig. 1C, *left*). Finally the shape of the action potential and the after-hyper-polarization were also subject to differences between the two classes of neurons. NFS cells had broader action potentials with a slow sloping after-hyper-polarization (Supplementary Fig. 1D, *left*). The FS cells on the other hand had narrower action potentials with a fast and “v-shaped” after-hyper-polarization enabling higher firing frequencies (Supplementary Fig. 1D, *right*). These parameters were taken together in the classification of FS vs. nFS.
